# Monoterpene Enrichments Have Positive Impacts on Soil Bacterial Communities and the Potential of Application in Bioremediation

**DOI:** 10.3390/plants10112536

**Published:** 2021-11-21

**Authors:** Dimitris Chalkos, Katerina Karamanoli, Despoina Vokou

**Affiliations:** 1 Department of Ecology, School of Biology, Aristotle University of Thessaloniki, GR-54124 Thessaloniki, Greece; chalkosd@gmail.com; 2 Laboratory of Agricultural Chemistry, School of Agriculture, Faculty of Agriculture Forestry and Natural Environment, Aristotle University of Thessaloniki, GR-54124 Thessaloniki, Greece; katkar@agro.auth.gr

**Keywords:** aromatic plants, biodegradation, cineol, fenchone, microbial succession, phrygana, pinene, soil respiration

## Abstract

We study here how soil bacterial communities of different ecosystems respond to disturbances caused by enrichments with monoterpenes that are common essential oil constituents. We used fenchone, 1,8-cineol and α-pinene, and soils from phrygana, a typical Mediterranean-type ecosystem where aromatic plants abound, and from another five ecosystem types, focusing on culturable bacteria. Patterns of response were common to all ecosystems, but responses themselves were not always as pronounced in phrygana as in the other ecosystems, suggesting that these enrichments are less of a disturbance there. More specifically, soil respiration and abundance of the bacterial communities increased, becoming from below two up to 16 times as high as in control soils (for both attributes) and remained at high levels as long as these compounds were present. Bacteria that can utilize these three compounds as substrates of growth became dominant members of the bacterial communities in the enriched soils. All changes were readily reversible once monoterpene addition stopped. Bacteria with the ability to utilize these monoterpenes as carbon sources were found in soils from all ecosystems, 15 strains in total, suggesting a rather universal presence; of these, six could also utilize the organic pollutants toluene or *p*-xylene. These results suggest also potential novel applications of monoterpenes in combating soil pollution.

## 1. Introduction

Bacterial abundance and diversity are very high in soil ecosystems [1,2], with a wide range of environmental factors, both biotic and abiotic, determining what will appear, where, and when [3,4,5]. Soil bacteria participate in fundamental processes such as degradation of organic matter and nutrient cycling, hence, changes in the structure of their communities affect ecosystem functioning and services directly or indirectly [6,7]. Soil bacteria may also alter soil structure [8], enhance soil fertility [9,10], neutralize environmental pollutants [11,12,13], promote plant growth and vigour [14,15,16], regulate greenhouse gas fluxes between the soil and the atmosphere [17,18], and participate in several other important processes [19,20].

Bacterial diversity is controlled primarily by soil parameters and is not related to factors that have been proven to affect the biodiversity of plants and animals [2]. Vegetation plays an important role; several studies showed that members of the rhizosphere microbial communities vary depending on the plant species participating in the aboveground vegetation and their chemistry [21,22]. Various plant ingredients, including volatile secondary metabolites such as the essential oil constituents of aromatic plants that abound in the Mediterranean environment arrive at the soil through various avenues [23,24], exerting their effects on the rhizosphere and other soil microbes.

Essential oils are mixtures of energy-rich [25], low molecular-weight compounds, of the chemical family of terpenes (isoprenoids), belonging primarily to two major groups: the C-10 monoterpenes and the C-15 sesquiterpenes (including their respective oxygenated derivatives). Essential oils are still present at high concentrations in the litter of aromatic plants; values of more than 4 mL per 100 g dry weight were reported for newly fallen litter [26]. Once in the soil environment, they are subject to transformations, many of which are mediated by microorganisms [27,28]. Essential oils have a multifaceted biological activity [29] and play important roles in plant-animal, plant-microbe, and plant-plant interactions [30,31,32], directly or indirectly [33]. Thoroughly documented is the antimicrobial activity of essential oils and of their individual constituents against a large variety of microorganisms, most of which are human, animal, or crop pathogens [34,35,36]. Despite this widely known antimicrobial activity, several studies showed that essential oils stimulate soil metabolism, increase bacterial biomass [37,38,39,40], enhance the activity of some soil enzymes [41,42] and are readily degraded by bacteria [43,44,45] or biotransformed by bacterial enzymes [46]. It was reported that microbes associated with aromatic plants can also degrade organic pollutants such as polychlorinated biphenyls (PCBs) and, therefore, they have a potential application in cleaning up contaminated soils [47].

In the current study, we explore the response of soil microbial communities from different ecosystem types to enrichments with three monoterpenes, which are common constituents of essential oils. Earlier studies with soils from Mediterranean-type ecosystems, where aromatic plants are main components of their vegetation, showed that essential oils and their ingredients increased soil respiration and bacterial abundance manifold [37,38,43] and that they also changed structural features of the community of culturable bacteria, with one or very few strains becoming highly dominant. These strains were both resistant to the compounds offered and able to use them as substrates of growth [39]. Focusing on culturable bacteria and using the same methodology as in these studies, we explore here the hypothesis that coexistence of microbes with aromatic plants matters and, hence, patterns of response of the soil communities from different ecosystems to enrichments with essential oil constituents will not be the same. We also examine if bacteria having the enzymatic machinery to catabolize such compounds, which are widely assumed to have antimicrobial properties, can also catabolize low molecular weight pollutants originating from organic sources such as the aromatic hydrocarbons toluene and *p*-xylene, which are described as priority pollutants [48].

We note that, for the first time, soil communities of six different ecosystem types were subjected concurrently to essential oil ingredients and monitored through time. Results provide insight on the effects of essential oils on processes related to the decomposition of aromatic plants in their natural environment and elsewhere, on the distribution of bacteria that are able to use their ingredients as substrates of growth and on potential applications of monoterpenes in bioremediation and sustainable agriculture.

## 2. Results

The soils of the six ecosystem types that we examined can be divided into two groups based on their carbon and nitrogen content (Table 1): of high and low nutrient status. Soils of the first group originated from forests (mixed deciduous, oak and riparian), whereas of the second group from non-forest ecosystem types (the typical Mediterranean-type ecosystem of phrygana, the sandy shore and the desert). Soils of the latter group had similar concentrations of nitrogen and organic carbon that may be more than an order of magnitude lower than in forest soils. The oak forest soil, which unlike the other soils lacked high participation of sand particles, was the richest of all in organic carbon and nitrogen.

Assays that were applied to the main culturable bacteria that were isolated from the soil samples of the different treatments and results taken are given in Tables S1 and S2. Of the bacterial isolates, 15 taxa belonging to 12 genera could be identified according to the Biolog identification system (Table 2). Some were confined in only one ecosystem, as is the case of *Micrococcus luteus* or *Variovorax paradoxus* that were encountered only in the riparian forest. Other strains proved with a wider occurrence, as is the case of *Micrococcus diversus* that was isolated from the mixed deciduous and oak forests, or *Tsukamurella inchonensis* that was isolated from all three non-forest ecosystem types and the oak forest.

Results on the effects of the three compounds, when added to soil samples from the six ecosystem types, are given here and in the Supplement. We provide here results for the oak forest, as a representative of the forest types, and for phrygana, the typical Mediterranean-type ecosystem, where aromatic plants are abundant, as a representative of the non-forest ecosystem types. For all other ecosystems, results are provided in the Supplement (Figures S1–S3).

**Figure 1:**
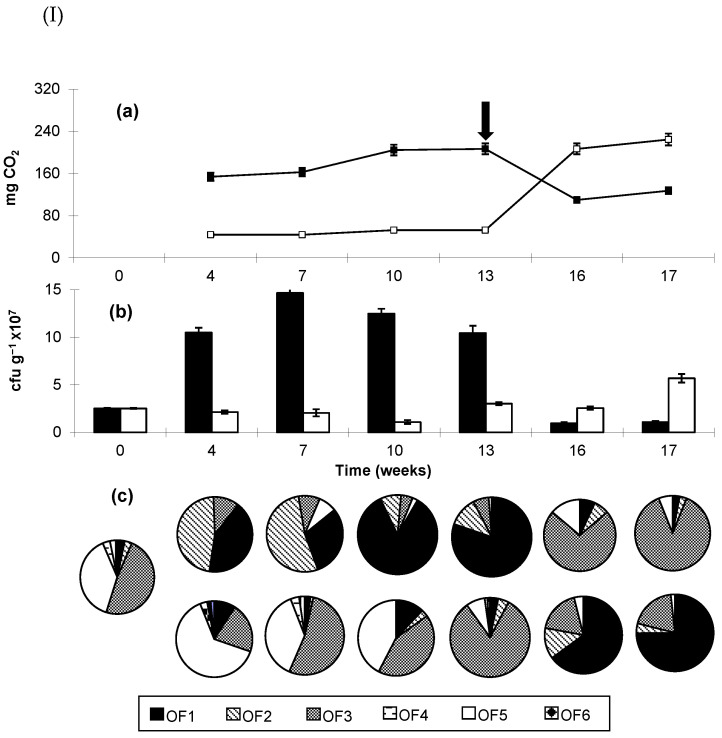

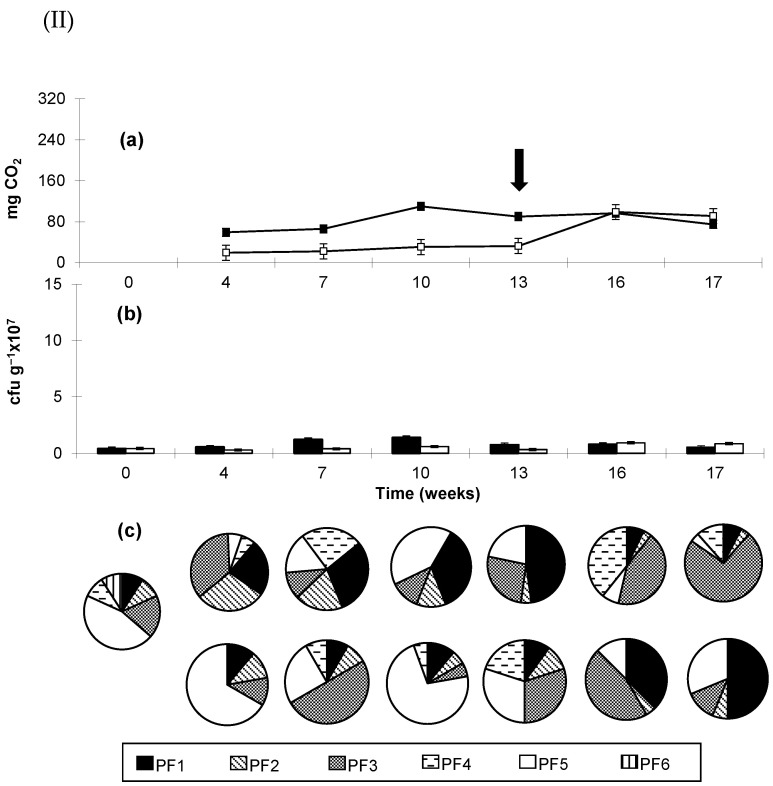
Results regarding (**a**) soil respiration (average ± se), (**b**) abundance of the community of culturable bacteria (average ± se) and (**c**) relative participation of the isolated main bacterial strains in enriched with fenchone (F) and in control soil samples, from (**I**) an oak forest (O) and (**II**) phrygana (P). Fenchone was added every week. Measurements for (**b**,**c**) were taken at the start of the experiment and on the 4th, 7th, 10th, 13th, 16th, and 17th week; measurements for (**a**) started on the 4th week. At the end of the 13th week, as indicated by an arrow, fenchone addition was reversed: control samples became the enriched ones and vice versa. In (**a**,**b**), white colour corresponds to control soil samples and grey to enriched ones. In (**c**), the upper row corresponds to treated samples and the lower to control samples. For the bacterial codes, see note 1 under Table 2.

Weekly addition of fenchone in the soil samples resulted in soil respiration higher than in control samples throughout the experiment, irrespective of ecosystem type (Figure 1a and Figure S1a, Table S3). The highest values of CO_2_ release were recorded in forest soils. Increases in soil respiration were always paralleled by significant increases in abundance of the culturable bacteria (Figure 1b and Figure S1b, Table S3); for phrygana, increase in abundance (Figure 1(IIb)) was less pronounced than for the other ecosystem types. Abundance of culturable bacteria and relative participation of each of the main strains among them in the soils at time 0 (Figure 1c and Figure S1c) correspond to measurements right after addition of the compounds tested in the soil. Monitoring of the relative participation of the main culturable bacteria showed that it changed with time, progressing in different directions for the enriched and the control samples (Figure 1c and Figure S1c, Table S4), with different bacteria becoming dominant. In the treatments with fenchone, dominance of some strains reached overwhelming proportions, as is the case of the oak forest (Figure 1(II)), in the soil of which relative participation of strain OF1 was more than 75%, ten weeks after the start of the experiment. The dominant bacterial strains (OF1, MF1, RF1, PF1, SF2 and DF2) in the soil samples enriched with fenchone (Figure 1 and Figure S1), when isolated, could grow uninhibited in the presence of this compound and were also capable of using it as a substrate of growth (Table 2).

**Scheme 1:**
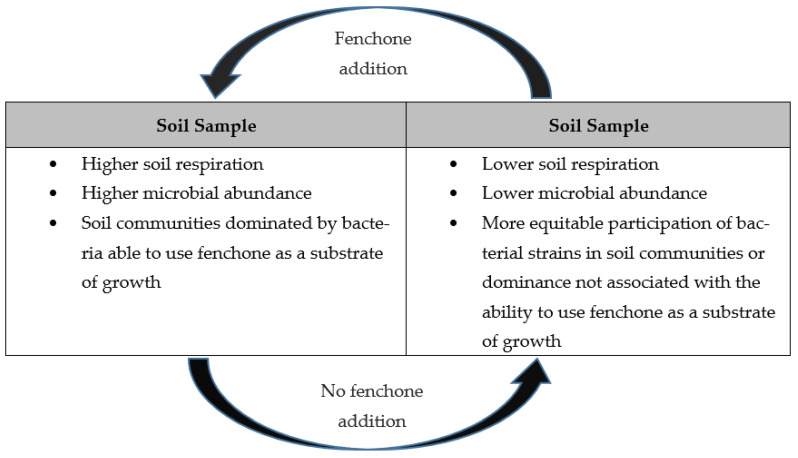
Representation of the cycle of events associated with presence or absence of fenchone.

The effect of fenchone on these structural features of the soil bacterial communities was readily reversible. When addition of fenchone came to a stop, there was a rapid decrease in the participation of the highly dominant strains; accordingly, addition of fenchone to the formerly control samples led to a similarly fast increase of the same strains that were previously dominant in the enriched samples (Figure 1 and Figure S1). Such rapid reverse changes were also recorded for soil respiration and bacterial abundance; they were least pronounced in the case of phrygana (Figure 1(II)). The overall effect of fenchone, as revealed from this study, on soil microbial communities is visualized in Scheme 1.

**Figure 2:**
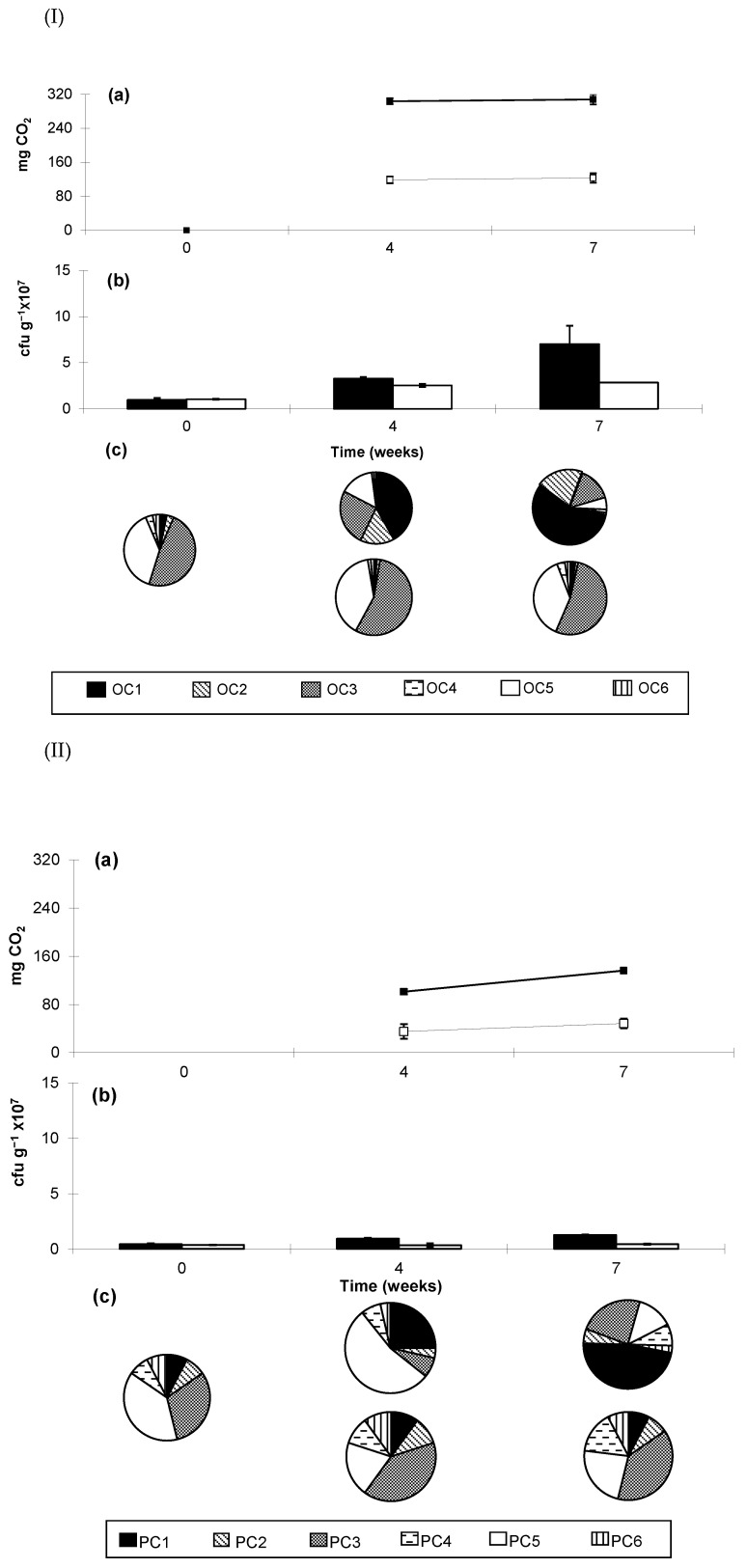
Results regarding (**a**) soil respiration (average ± se), (**b**) abundance of the community of culturable bacteria (average ± se) and (**c**) relative participation of the isolated main bacterial strains in enriched with 1,8-cineol (C) and in control soil samples, from (**I**) an oak forest (O) and (**II**) phrygana (P). 1,8-Cineol was added every week. Measurements for (**b**,**c**) were taken at the start of the experiment and then on the 4th and 7th week; measurements for (**a**) started on the 4th week. In (**a**,**b**), white colour corresponds to control soil samples and grey to enriched ones. In (**c**), the upper row corresponds to treated samples and the lower to control samples. For the bacterial codes, see note 1 under Table 2.

Enrichment of soil samples with 1,8-cineol resulted in responses similar to those of the fenchone-enrichment. In the presence of cineol, soil respiration (Figure 2a and Figure S2a, Table S5) increased and, similarly, abundance of culturable bacteria (Figure 2b and Figure S2b, Table S5). As with fenchone, the highest values of CO_2_ release were recorded in soils from forests. The relative participation of the main culturable bacteria in the soils changed with time (Figure 2c and Figure S2c, Table S6), with some strains profiting from the presence of cineol and dominating the community; these were different to the ones dominating the control samples. Changes in abundance of the culturable bacteria were more pronounced in soil samples from the mixed deciduous forest (Figure S2(II)). Dominance culminated in samples from this ecosystem, in which one bacterial strain (MC1) had a relative participation of more than 90% (Figure S2(IIc)). Dominance was lowest in phrygana (Figure 2(IIc)), where the highest relative participation recorded (PC1) was less than 50%. In all cases, bacterial strains dominating the communities enriched with cineol (strains OC1, MC1, RC1, PC1, SC2 and DC2) (Figure 2c and Figure S2c), when isolated, could grow uninhibited in the presence of this compound and were also capable of using it as a substrate of growth (Table 2).

**Figure 3:**
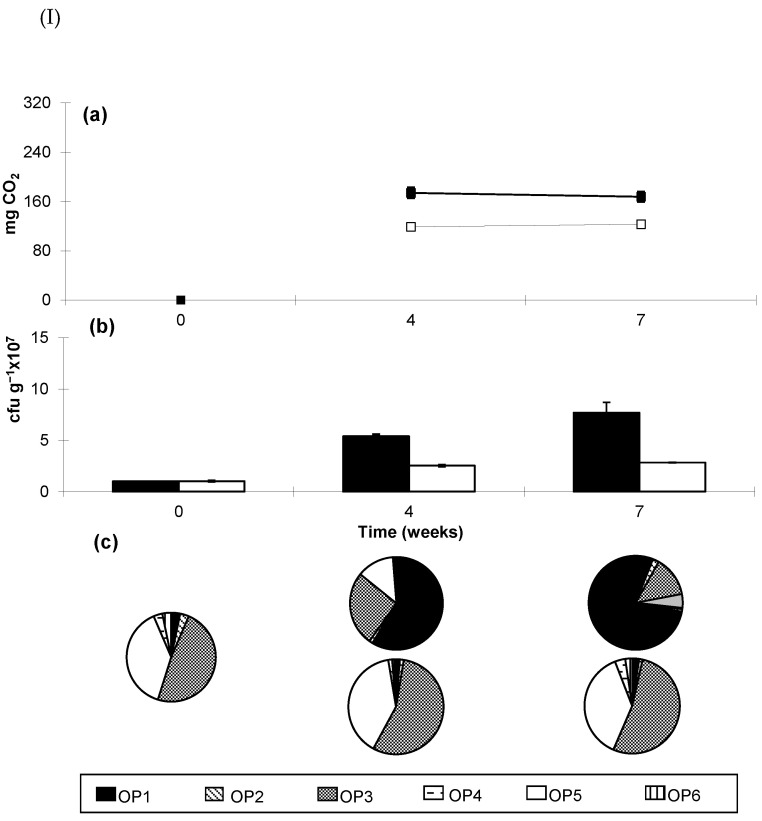

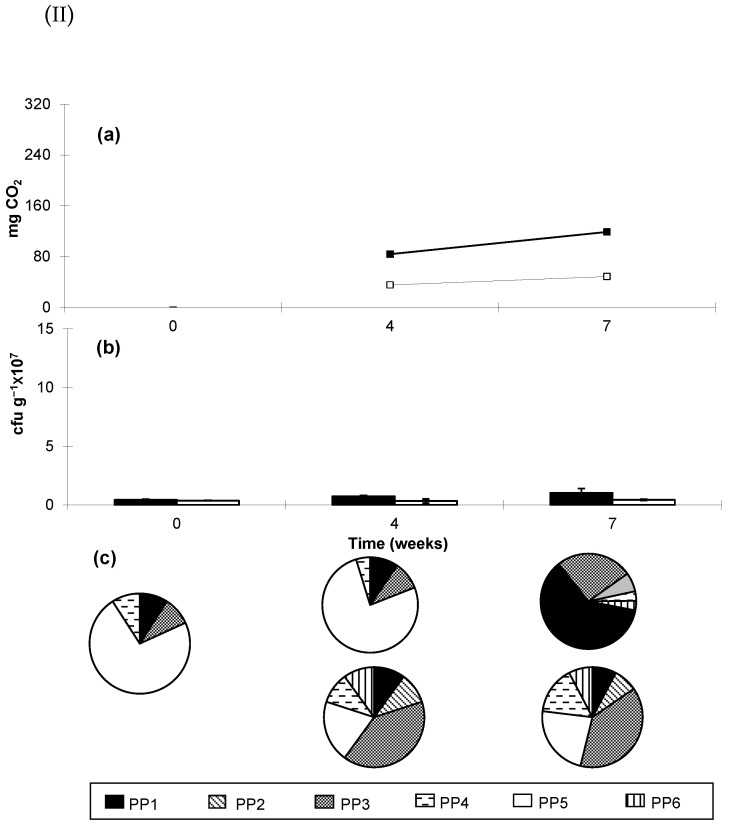
Results regarding (**a**) soil respiration (average ± se), (**b**) abundance of the community of culturable bacteria (average ± se) and (**c**) relative participation of the isolated main bacterial strains in enriched with α-pinene (P) and in control soil samples, from (**I**) an oak forest (O) and (**II**) phrygana (P). α-Pinene was added every week. Measurements for (**b**,**c**) were taken at the start of the experiment and then on the 4th and 7th week; measurements for (**a**) started on the 4th week. In (**a**,**b**), white colour corresponds to control soil samples and grey to enriched ones. In (**c**), the upper row corresponds to treated samples and the lower to control samples. For the bacterial codes, see note 1 under Table 2.

The effects of pinene on soil respiration (Figure 3a and Figure S3a, Table S7), abundance of culturable bacteria (Figure 3b, Figures S3b and S7, Table S7) and relative participation of the main strains among them (Figure 3c and Figure S3c, Table S8) were similar to those of the other two compounds, but not always as pronounced. The highest values of soil respiration and abundance were again recorded in forest soils (Figure 3(Ib) and Figure S3(Ib,IIb)), but abundance attained high values also in the desert soil samples (Figure S3(IVb)); it remained quite low in phrygana (Figure 3(IIc)). Regarding the relative participation of the main culturable strains of the bacterial community, pinene did not always trigger marked differences. For instance, the same bacteria were the dominant ones in both the control and the enriched soil samples of the mixed deciduous and the riparian forests (Figure S3(Ic,IIc). In all cases, bacterial strains dominating the communities enriched with pinene (strains OP1, MP3, RP3, PP1, SP2 and DP1) (Figure 3c and Figure S3c), when isolated, could grow uninhibited in the presence of this compound and were able to use it as a substrate of growth (Table 2).

Of the identified bacteria, none was inhibited in the presence of pinene, even at the highest rate tested (15 μL). This was also the case for nine bacteria in the presence of fenchone and for six bacteria in the presence of cineol (Table 2 and Table S9). Most sensitive to fenchone was *Pseudomonas caricapapayae*; its growth was completely inhibited not only at the highest but also at the intermediate rate (10 μL). The same strain was also the most sensitive to cineol. All bacteria that were resistant to cineol were also resistant to fenchone. All bacteria that were fully resistant to cineol could also utilize it as a substrate of growth (Table 2). Similarly, all bacteria fully resistant to fenchone had the ability of utilizing it, except for *Brevibacterium mcbrellneri*, but this was not the case for pinene, as nine fully resistant strains proved incapable of using it as a carbon source. In total, 11 identified bacteria could use at least one of the monoterpenes offered as a substrate of growth; they belong to the bacillus (7 strains) and coccus (4 strains) types and to both Gram-positive (5 strains) and Gram-negative bacteria (6 strains) (Table 2 and Table S2). As in the experiments of substrate utilization, bacteria were offered the three monoterpenes as a sole carbon source, when oxidization was detected, it signified the ability of bacteria to degrade them. Of the 11 monoterpene-degrading strains, six could also degrade at least one of the two xenobiotics examined: *C. nitrilophilus, P. stewartii stewarti* and *R. rhizogenes* had this ability for both toluene and *p*-xylene, whereas *R. ruber*, *B. glumae* and *M. diversus* for only *p*-xylene; none had this ability for only toluene.

## 3. Discussion

Fenchone, a bicyclic monoterpenic ketone, is a main constituent of many essential oils such as of the genera *Thuja* [49], *Lavandula* [39], *Foeniculum* [50], etc. In an experiment investigating the antibacterial activity of 20 oxygenated monoterpenes, it was reported that fenchone was one of the few compounds that affected a large number among the 63 bacterial strains examined [51]. Others reported the antimicrobial activity of the essential oil of *Lavandula stoechas*, having fenchone as a main ingredient, against six bacterial and two fungal strains [52], or attributed the antimicrobial activity of essential oils from representatives of *Commiphora* (Burseraceae) to the high concentration of oxygenated monoterpenes including fenchone [53]. In a different direction, when *L. stoechas* essential oil or its main constituent fenchone were added to soil from a Mediterranean ecosystem, soil respiration increased, and certain bacterial strains proliferated [39].

1,8-Cineole, a cyclic monoterpenic ether, occurs as a main ingredient in essential oils of many aromatic plants, such as representatives of the genus *Eucalyptus* [54], *Lavandula* [36,39], *Rosmarinus* [27,39,55], *Salvia* [39,56], etc. Antimicrobial activity has been repeatedly reported for this compound [57,58], but not always very strong [36,54,55,59]. In our experiments, here, it proved with slightly higher inhibitory activity than fenchone. When cineol was added to soil samples from a Mediterranean ecosystem, soil respiration increased manifold [37].

α-Pinene, a bicyclic monoterpene, occurs abundantly in conifers [60,61] and is a main constituent in the essential oils of many other plants such as the genera *Rosmarinus* [36], *Salvia, Sideritis* [62], etc. It possesses antimicrobial properties [55,63], very often weaker than the oxygenated compounds [36,58].

The stimulation of soil respiration in the presence of the three essential oil constituents that we examined ranged from 1.4 times as high as in the control (in the soil from the oak forest treated with pinene) up to 15 times (in the soil from the sandy shore treated with fenchone). This stimulation is directly linked to the activation of the soil bacterial community that, simultaneously, increased in abundance; its culturable part became 2.7 and 7.7 times as high as in the control, respectively, in the soils of the above two treatments. Changes in the two attributes were not always of corresponding magnitude; for instance, for the fenchone enrichments, the minimum increase in bacterial abundance was recorded in the soil from phrygana (1.9. times as high as in the control), whereas the maximum in the soil of the mixed deciduous forest (16 times). These responses are in accordance with results that were reported in the past under comparable conditions [37,38,39,43].

There was a variety of responses of the soil bacterial communities to enrichments with fenchone, cineol or pinene with respect to structural features. For instance, a single strain could dominate the soil bacterial community of an ecosystem under all three types of enrichment, as is the case of *Rhodococcus ruber* becoming dominant in the oak forest soil, *Pantoea stewartii stewartii* (formerly *Erwinia*) in the sandy shore soil, and *Corynebacterium nitrilophilus* in the phrygana soil. In other cases, one strain could dominate the soil communities of more than one ecosystem type, as is *Burkholderia glumae* becoming dominant in soils from both the mixed deciduous and the riparian forests when enriched with fenchone or cineol. The prolonged experiment with fenchone also showed that changes in all types of response examined were fully reversible after enrichment stopped. This indicates highly dynamic microbial communities readily responding to perturbations in their environment.

In a typical Mediterranean ecosystem such as that of phrygana, soil enrichment with essential oils or their constituents may be considered as a natural phenomenon in the yearly cycle of events; it may happen even twice every year, when the seasonal dimorphic shrubs, among which many aromatic plants such as *Thymus capitatus, Satureja thymbra, Teucrium polium, Origanum onites* [26,64] drop their leaves. However, the fast and reversible response of microbial communities to monoterpenes is not an adaptation of soil microbes to the aromatic plants of this environment; it seems to be a rather universal feature. Bacterial strains able to degrade essential oil constituents have been isolated from soils supporting plants that produce them [65], but not exclusively from there [28,66]. This widespread degrading ability can be explained as resulting from the structural analogy of the three compounds to molecules that the microbes had a former experience of, as several non-aromatic plants emit compounds regularly found in essential oils. For instance, *Quercus ilex* emits α-pinene, limonene and other monoterpenes, at rates higher by one order of magnitude in summer than in winter [67]. Additionally, a large variety of plants emit isoprene, a hemiterpene that is considered the monomer of all isoprenoid compounds; its emissions are huge, comparable to those of methane [68]. Soil can act as a sink for atmospheric isoprene as bacteria in it are able to degrade it [69]. All this suggests that bacteria in the soils that we examined could have encountered in their environment the compounds that we offered or other structurally related, in forms other than of essential oil constituents, and that some have acquired the enzymatic machinery to degrade them. In fact, bacteria with isoprene degrading ability such as *Rhodococcus* representatives [70,71] were also found able to biotransform monoterpenes [28,46]. Similarly, in this study, we found a *Rhodococcus* strain, dominant in the treated oak forest soil samples, to be able to use all three monoterpenes offered as substrates of growth.

The two organic pollutants that we examined, toluene and *p*-xylene, could be used as substrates of growth by bacteria that could also use at least one of the three essential oils constituents tested. Among these bacteria is a strain belonging to the genus *Burkholderia* (formerly *Pseudomonas*), members of which are known degraders of these two pollutants [72]. Earlier studies showed that monoterpenes stimulate the biodegradation of xenobiotics such as dichlorophenol [12], poly-chlororinated biphenyls and other compounds [47,73,74] by indigenous soil microorganisms. Among these microorganisms are representatives of the genera *Rhodococcus, Burkholderia* and *Corynebacterium* [74], strains of which were also found here to be able to utilize these two organic pollutants as substrates of growth. Additionally, predominant hydrocarbon-degrading bacteria in the natural environment include strains of *Pseudomonas* and *Corynebacterium* that were also detected in our samples [75]. As a result of this stimulating activity on xenobiotic degraders, aromatic plants or monoterpene amendments were proposed as a strategy for removing organic contaminants from the soil [47]. In fact, plants have been used for efficient remediation of petroleum hydrocarbon contaminated soils [75]. Our findings show the strong influence that essential oil ingredients exert on the soil microbial community, favouring bacterial consortia that can degrade these compounds and also organic pollutants. They suggest, therefore, effectiveness of such strategies and promising novel applications for aromatic plants, if the latter prove to have effects similar to those of their ingredients that we examined here.

Patterns of change with monoterpene enrichment schematically represented here for fenchone, but valid for many other related compounds (non-published data), are common to soils of all six ecosystem types. However, there are quantitative differences. The degree to which essential oil constituents can activate and increase the size of the soil bacterial community depends on the system capacity to support it, which is related to the soil nutrient status. Forest soils, which were richer in carbon and nitrogen than the non-forest ones, generally exhibited higher values of soil respiration and bacterial biomass even in the control samples. In a number of cases, responses of soil bacteria from phrygana were not as pronounced as from the other ecosystem types. More specifically, increases in abundance in the presence of any of the three compounds were the smallest among the six ecosystem types; the dominance of one bacterial strain never attained the very high levels reached in some other ecosystem types; increase in soil respiration was always moderate; and when the addition of the compounds to the treated soil samples stopped and started instead to the previous control samples, there were no notable differences among the enriched and control soil samples, in contrast to the other ecosystem types. These quantitative differences to the common response patterns may be associated with the fact that the soil bacterial community in phrygana is more regularly exposed to such compounds than the soil communities of the other systems.

Given the impacts of the compounds that we examined on the soil bacterial communities, we propose the following scheme for the decomposition in the Mediterranean environment, where aromatic plants abound, as a hypothesis to be explored in future studies: Bacteria that can consume the energy-rich essential oil constituents are the primary colonizers of the litter of aromatic plants. After depleting it from materials potentially toxic to other soil organisms, they transform it into a safe substrate of growth. Essential oils would thus regulate the successional process in the detritus food chain, wherever they are present, either naturally or artificially. As a follow-up of this study, the entire spectra of the soil microbial communities should also be examined, both their culturable and non-culturable parts, so as to have information on all changes in their structure that are triggered by enrichments. Additionally, experiments using together two or more essential oil ingredients, applied at different concentrations, will provide information on the way that they act when in mixture and allow predictions regarding the activity of essential oils of different compositions. At an applied level, experiments are needed to verify that essential oils containing the tested monoterpenes and aromatic plants producing them enhance the potential of soil microbial communities to degrade the pollutants that we examined. Positive results with the aromatic plants would be very important as they would ensure economic viability of the application. Else, strategies employing special techniques [76] would be required for these monoterpenes to be applied for bioremediation purposes and in sustainable agriculture.

## 4. Materials and Methods

### 4.1. Soil Samples

Samples were collected from the upper soil layer (<20 cm) of the following six ecosystem types: (i) phrygana, from the island of Naxos, in the Cyclades group of the Aegean Sea, (ii) sandy coast, from the island of Skiathos, in the Sporades group of the Aegean Sea, (iii) desert, from Dubai, United Arad Emirates and also from three forest types from Chalkidiki peninsula, in northern Greece, more specifically from (iv) an oak forest, (v) a mixed deciduous forest, and (vi) a riparian forest. The geographic coordinates and altitude of the areas where sampling took place are given in Table 1. Aromatic plants are abundant in phrygana, which is a major Mediterranean-type ecosystem [37], but their participation in the other ecosystems is very low to none; there were no aromatic plants in any of the other five, within a radius of at least 50 m from the sampled sites.

Soil samples were manually treated to remove gravels and roots and were stored at 4 °C until use. Soil texture was determined according to [77].

### 4.2. Carbon and Nitrogen Analyses

Organic carbon and total nitrogen were estimated in three replicate soil samples for each of the six ecosystem types. For organic carbon, the wet oxidation method [78] was used. In brief, samples (0.1 g) were oxidized using K_2_Cr_2_O_7_–H_2_SO_4_ solution (1:1 *v*/*v*); total organic carbon was determined by titration with ferrous ammonium sulphate solution. For the total nitrogen content, the Kjedahl method [79] was used.

### 4.3. Essential Oil Constituents

The essential oil constituents that we applied were commercially supplied (Sigma-Aldrich Chemical Co. St. Louis, MO, USA). These were (1*R*)-(−)-fenchone (>98%), 1,8-cineol (99%) and (1*R*)-(+)-α-pinene (>99%), referred in the text as fenchone, cineol and pinene, respectively. They were stored at 4 °C until use.

### 4.4. Experimental Design and Conditions

The experimental design to examine the effects of fenchone, cineol and pinene on soil bacteria and soil metabolism, as expressed by soil respiration, was as follows: Three sets of experiments were run, corresponding to each of the three compounds examined. Every set consisted of soil samples from each of the six ecosystem types enriched with the compound under study and of the corresponding control samples, all with three replicates.

The method that we applied for these experiments was described before [39]. In brief, soil samples (150 g dry weight) from each ecosystem type were put in airtight containers (aluminium cylinders of 860 cm^3^ capacity); 30 mL of distilled water were added to them and a 50-mL beaker with 20 mL 1M KOH was placed in each container, on the top of the soil. Apart from the control and the treated soil samples, there were also containers with only the beaker and KOH. Experiments were conducted at room temperature (24–29 °C). Given their magnitude, they were not concurrently run. Including six soil types, two types of enrichment (compound added or not), three compounds tested, and three replicates, a total of 108 (6 × 2 × 3 × 3) such containers were prepared and soil samples within them assessed.

The three compounds (fenchone, cineol, pinene) were repeatedly added into soil samples, according to the methodology previously described [37]. More specifically, 0.1 mL was added once every week for a total of seven weeks. We did additional explorations with fenchone so as (i) to have a better estimate of how permanent the induced changes are, and (ii) how reversible they are once the disturbance (i.e., the enrichment) stops. For (i) we extended the duration of the experiment for another six weeks (13 weeks, in total). For (ii), at the end of the 13th week, we reversed samples: we stopped adding fenchone to the till then treated soil samples and started adding it instead to the till then control samples; to the latter, we continued adding fenchone, on a weekly basis, for another four weeks.

#### 4.4.1. Soil Respiration

Soil respiration was first determined on the 4th week after the establishment of the experiment, and then every 3 weeks in both the control and the enriched soil samples. In the case of fenchone, it was additionally estimated on the 10th, 13th, 16th, and 17th week. We did not start taking measurements right from the beginning, because previous experiments showed that microbial responses are not always clear in the first few weeks after exposure for the first time to essential oils or their constituents [38]. CO_2_ released was estimated by titration with 0.1 M, as described in [39].

#### 4.4.2. Abundance of the Culturable Part of the Soil Bacterial Community

Using culture methods, we estimated the soil bacterial abundance at the start of the experiment (time 0) and then on the 4th and 7th week, in the control and the treated samples. In the case of fenchone, it was additionally estimated on the 10th, 13th, 16th, and 17th week. We used the soil dilution plate method and we counted bacterial colonies formed on a general-purpose medium in Petri dishes. We used dilutions from 10^−2^ to the dilution that produced less than 100 CFU per plate; This was often the case at 10^−4^, but it could be at 10^−5^ or even 10^−6^, mostly in the treated soils. Dilutions of the soil samples were plated onto nutrient agar (10 g tryptone peptone, 5 g yeast extract, 5 g NaCl, 15 g Bacto Agar, 1 L dH_2_O) and agar plates were incubated in the dark at 30 °C. We checked colony growth every 24 h and counted colonies after 72 h. There were three replicates for each dilution of each sample. Results are expressed as CFU g^−1^ of dry weight soil.

#### 4.4.3. Structural Features of the Soil Bacterial Community (Relative Participation of the Main Culturable Strains)

We studied the main culturable bacteria (six to seven) in the soils of the six ecosystem types focusing on how their contribution in the community changes with time and treatment. We targeted these bacteria to further examine if dominance in the treated soils can be explained in terms of resistance to and ability to use the monoterpenes offered as substrates of growth. We made use of Bergey’s Manual of Determinative Bacteriology [80] and the Biolog identification system (Biolog Inc., Hayward, CA, USA). We obtained pure cultures of the morphologically different colonies that were formed in Petri dishes, deriving from enriched and control soil samples. These cultures were maintained at 4 °C and transferred to new nutrient media at regular intervals, depending on their growth rate. Isolated bacterial stains were classified based on the following major characteristics: (i) colour of the colonies in the solid medium (visual observation), (ii) shape of cells (microscopic observation), (iii) Gram reaction, (iv) ability to form endospores (microscopic observation), (v) ability for oxidase and catalase production, and (vi) mobility (motility). For each pure culture, tests (iii) to (vi) were repeated twice giving identical results. Bacteria with same characteristics for these six parameters were classified as one strain. We estimated the relative participation of each of the main bacteria at the start of the experiment (time 0) and as time progressed for all treatments. Τhe relative participation of each strain under study was estimated after the number of the colonies it formed compared to the colonies of all main bacteria of the treatment. Isolated strains were given names consisting of two letters and a number. The first letter corresponds to the ecosystem where they were isolated from (M for mixed deciduous forest, O for oak forest, R for riparian forest, P for phrygana, S for sandy shore, and D for desert) and the second to the compound examined (F for fenchone, C for 1,8-cineol, and P for α-pinene). The number corresponds to each of the six (seven in the case of the mixed deciduous forest and fenchone) main bacterial strains that were isolated from the soil samples of the different treatments including the control. In all Figures, we use code names for bacteria.

With the Biolog system, individual bacterial strains can be identified according to their ability to use various carbon sources [81]. Depending on their response to Gram staining, isolated bacteria were grown in GP2 (for Gram-positive) or GN2 (for Gram-negative) Biolog plates. Each plate has wells containing one of 95 carbon sources (Table S1) and a redox dye (tetrazolium violet) that turns from colourless to purple when oxidized, thus showing metabolic activity of the inoculated bacterial strain [82]; the unique colour pattern resulting from oxidized and non-oxidized substrates is the metabolic fingerprint of the strain. After inoculation, the plates were incubated at 30 °C. Three Biolog plates were used for each strain examined. The metabolic fingerprint of each bacterial strain was determined by estimating the optical density at 590 nm and was then compared with records of the Biolog database for aerobic bacteria that includes 1568 taxa (Biolog Inc., Hayward, CA, USA). Similarity indices that were calculated after the reaction profiles of the strain under study and those in the database led to identifications of the unknown strains. Acceptable identifications were the ones with a similarity index of at least 0.50 for readings at 48–72 h [82].

#### 4.4.4. Resistance to Essential Oil Constituents

The 15 bacterial strains that could be identified after the Biolog system were tested in vitro for their resistance against fenchone, cineol and pinene, by use of the disk diffusion method. For this, 100 μL of a fresh overnight culture of each strain in 10 mL nutrient broth (Sigma-Aldrich Chemie GmbH, Taufkirchen, Germany) were plated onto Petri dishes containing nutrient agar, same as the one described in Section 4.4.2. A sterile filter paper (Whatman No. 1, 0.5 mm diameter) was placed at the centre of the dish. The filter paper was then impregnated with the compound tested (at 5, 10 and 15 μL) and the plate was sealed with parafilm. There were five replicates per treatment. Plates were incubated at 30 °C for 24 h, after which the width of the inhibition zone around the filter paper (in mm) was measured. Results are expressed as full, moderate, and low resistance. Full resistance corresponds to uninhibited growth at any of the three rates at which each compound was applied. Moderate resistance corresponds to an inhibition zone up to 10 mm at either of the two higher rates, whereas low resistance to an inhibition zone above 10 mm and below 30 mm. When the inhibition zone was larger than 30 mm, the specific strain was considered as having no resistance at all.

#### 4.4.5. Utilization of Essential Oil Constituents as Substrates of Growth

We examined the ability of the 15 identified bacterial strains to utilize fenchone, cineole and pinene as substrates of growth. For this, we made use of the MT2 plates of the Biolog system. We added fenchone, cineol and pinene, separately, in five replicate wells that did not contain any other carbon source. As in the case of plates in Section 4.4.3, the ability of bacteria to utilize each of these compounds as a carbon source was detected by the reduction of tetrazolium dye that was also added in each cell resulting in colour change when the substrate was oxidized. Wells resembling the colourless control were scored as negative, whereas those with a noticeable change of colour (light to deep purple) as positive; accordingly, bacteria were described as unable or able to utilize the essential oil constituents as substrates of growth. A deep purple colour means a fully oxidized substrate suggesting that it is a favourite carbon source for the bacterial strain under study; less preferred sources are consumed slowly or incompletely, leading to lighter colours [82]. For all strains examined, all five replicates had the same colour.

### 4.5. Utilization of Organic Pollutants as Substrates of Growth

We examined if bacterial strains able to use fenchone or cineol or pinene as a carbon source could also use toluene (99.8%) and *p*-xylene (≥99%) (Sigma-Aldrich Chemie GmbH, Taufkirchen, Germany). These low molecular weight aromatic hydrocarbons are common aromatic solvents and are also involved in the production and use of gasoline; they are considered as priority pollutants [48]. For these assays, we used the Βiolog MT2 plates, as in Section 4.4.5, and followed the procedure described there. The two compounds were applied at 100 mM, in five replicate wells. For all strains examined, all five replicates had the same colour.

### 4.6. Data Analysis

For each set of experiments, separate MANOVAs were run to test for differences in (i) soil respiration, (ii) size, and (iii) structural features of the bacterial communities. For the dependent variables (i) and (ii), enrichment was the independent variable and sampling time the repeated variable. For variable (iii), enrichment and relative participation of the main culturable strains were the independent variables and sampling time the repeated variable.

Data of substrate use and of resistance to fenchone, cineol and pinene, were analyzed with ANOVA. Means were compared using the LSD test and applying the Bonferroni adjustment.

Wherever average values of variables are presented, given are also the standard errors (se) of the estimations. All statistical analyses were performed using the STATISTICA software (ver. 7.06, Statsoft Inc., Tulsa, OK, USA).

## 5. Conclusions

Results of this study clearly demonstrate (i) that the essential oil constituents that we examined do not exert inhibitory effects on soil respiration, (ii) that they favour bacterial strains that are not only resistant to them but can also use them as substrate of growth, and (iii) that bacteria with such properties have a rather ubiquitous presence. At an applied level, as the examined essential oil ingredients enrich selectively the soil environment with microbes that can utilize both these metabolites and the xenobiotic contaminants as carbon sources, they have a potential application in combating soil pollution and in sustainable agriculture. Patterns of response to enrichment with monoterpenes are common to soil bacteria from all ecosystems examined, although responses themselves were not always as pronounced in phrygana, a typical Mediterranean-type ecosystem with aromatic plants among its main components, as in the other ecosystem types. This suggests that soil enrichments with these compounds in the Mediterranean environment are less of a disturbance than in other environments where aromatic plants are absent.

## Figures and Tables

**Table 1:** Soil texture, organic matter, and organic nitrogen content (average ± se) of soil samples from the six ecosystem types that were used in the experiments, and coordinates and altitude of the sampling sites.

Coordinates/Altitude of the Sampling Sites	Ecosystem Type (Abbreviation)	Soil Texture	Total Organic Carbon (%)	Total Nitrogen (%)
40°25′52″ N, 23°33′51″ E/767 m	Oak forest (O)	Clay loam	8.20 ± 0.12	0.35 ± 0.08
40°25′26″ N, 23°23′13″ E/590 m	Mixed deciduous forest (M)	Sandy clay loam	4.10 ± 0.15	0.15 ± 0.04
40°26′27″ N, 23°24′13″ E/508 m	Riparian forest (R)	Sandy clay	3.90 ± 0.10	0.10 ± 0.05
37°03′06″ N, 25°22′43″ E/52 m	Phrygana (P)	Sandy clay	0.70 ± 0.05	0.05 ± 0.02
39°08′57″ N, 23°24′16″ E/2 m	Sandy shore (S)	Sandy	0.70 ± 0.05	0.03 ± 0.01
25°04′33″ N, 55°15′18″ E/30 m	Desert (D)	Sandy	0.60 ± 0.03	0.02 ± 0.01

**Table 2:** Isolated bacterial strains and their occurrence, identification, resistance to the monoterpenes added, and ability to use fenchone (F), 1,8-cineol (C), α-pinene (P), toluene and *p*-xylene as substrates of growth; for the latter two compounds, this ability was tested only for bacteria that could use at least one of the three monoterpenes. Bacteria were isolated from the soils of mixed deciduous forest (M), oak forest (O), riparian forest (R), phrygana (P), sandy shore (S), and desert (D) ^1^. Whenever codes are in bold, it means that the corresponding bacteria were dominant, making more than half of all colonies of the main culturable bacteria that were isolated from the soil samples of the specific treatment. The symbol (+) means that the isolated bacteria had the ability to use the compound offered, (-) that they did not, whereas (++) means that this ability was strong. For full description of the levels of the different responses, see Materials and Methods.

Occurrence and Coding of the Bacteria Isolated and Identified	Identified Taxa ^2^	Resistance to Essential Oil Constituents		Use as Growth Substrates of			
				Essential Oil Constituents		Aromatic Hydrocarbons	
Mixed deciduous forest: MF3, MC3, **MP3**	*Brevibacterium* *mcbrellneri*	Fenchone: 1,8-Cineol: α-Pinene:	full moderate full	Fenchone: 1,8-Cineol: α-Pinene:	- - +	Toluene: *p*-Xylene:	- -
Phrygana: PF4, PC4, PP4	*Burkholderia* *gladioli*	Fenchone: 1,8-Cineol: α-Pinene:	low moderate full	Fenchone: 1,8-Cineol: α-Pinene:	- - -		
Mixed deciduous forest: **MF1**, **MC1**, MP1; Riparian forest: **RF1**, **RC1**, RP1; Phrygana: PF5, PC5, PP5	*Burkholderia* *glumae*	Fenchone: 1,8-Cineol: α-Pinene:	full full full	Fenchone: 1,8-Cineol: α-Pinene:	++ ++ -	Toluene *p*-Xylene	- ++
Desert: DF5, DC5, DP5	*Buttiauxella* *gaviniae*	Fenchone: 1,8-Cineol: α-Pinene:	moderate low full	Fenchone: 1,8-Cineol: α-Pinene:	- - -		
Desert: **DF2**, **DC2**, DP2	*Corynebacterium* *lypophylophlavum*	Fenchone: 1,8-Cineol: α-Pinene:	full full full	Fenchone: 1,8-Cineol: α-Pinene:	++ ++ -	Toluene *p*-Xylene	- -
Phrygana: **PF1**, PC1, **PP1**	*Corynebacterium* *nitrilophilus*	Fenchone: 1,8-Cineol: α-Pinene:	full full full	Fenchone: 1,8-Cineol: α-Pinene:	+ + ++	Toluene *p*-Xylene	+ ++
Mixed deciduous forest: MF2, MC2, MP2; Oak forest: OF2, OC2, OP2	*Micrococcus* *diversus*	Fenchone: 1,8-Cineol: α-Pinene:	full full full	Fenchone: 1,8-Cineol: α-Pinene:	+ + -	Toluene *p*-Xylene	- ++
Riparian forest: RF3, RC3, **RP3**	*Microccocus* *luteus*	Fenchone: 1,8-Cineol: α-Pinene:	moderate low full	Fenchone: 1,8-Cineol: α-Pinene:	- - +	Toluene *p*-Xylene	- -
Sandy shore: SF4, SC4, SP4	*Neisseria* *canis*	Fenchone: 1,8-Cineol: α-Pinene:	full low full	Fenchone: 1,8-Cineol: α-Pinene:	+ - -	Toluene *p*-Xylene	- -
Sandy shore: **SF2**, **SC2**, **SP2**	*Pantoea* *stewartii* *stewartii*	Fenchone: 1,8-Cineol: α-Pinene:	full full full	Fenchone: 1,8-Cineol: α-Pinene:	+ + +	Toluene *p*-Xylene	+ ++
Mixed deciduous forest: MF6, MC5, MP5; Oak forest: OF5, OC5, OP5; Riparian forest: RF5, RC5, RP5	*Pseudomonas* *caricapapayae*	Fenchone: 1,8-Cineol: α-Pinene:	No resistance No resistance full	Fenchone: 1,8-Cineol: α-Pinene:	- - -		
Desert: DF1, DC1, **DP1**	*Rhizobium* *rhizogenes*	Fenchone: 1,8-Cineol: α-Pinene:	low low full	Fenchone: 1,8-Cineol: α-Pinene:	- - ++	Toluene *p*-Xylene	+ +
Oak forest: **OF1**, **OC1**, **OP1**	*Rhodococcus* *ruber*	Fenchone: 1,8-Cineol: α-Pinene:	full full full	Fenchone: 1,8-Cineol: α-Pinene:	++ + ++	Toluene *p*-Xylene	- +
Oak forest: OF3, OC3, OP3; Desert: DF3, DC3, DP3; Sandy shore: SF3, SC3, SP3; Phrygana: PF3, PC3, PP3	*Tsukamurella* *inchonensis*	Fenchone: 1,8-Cineol: α-Pinene:	low moderate full	Fenchone: 1,8-Cineol: α-Pinene:	- - -		
Riparian forest: RF2, RC2, RP2	*Variovorax* *paradoxus*	Fenchone: 1,8-Cineol: α-Pinene:	full low full	Fenchone: 1,8-Cineol: α-Pinene:	+ --	Toluene *p*-Xylene	- -

^1^ The first letter of the coding system for the bacterial strains corresponds to the ecosystem from where they were isolated and the second to the compound examined; the number corresponds to each of the six (seven in the case of the mixed deciduous forest and fenchone) main bacterial strains that were isolated from the soil samples of the different treatments, which were also main strains in the control soil samples. ^2^ According to the Biolog identification system.

## Data Availability

The data presented in this study are available on request from the corresponding author.

## Supplementary Materials

The following are available online at https://www.mdpi.com/article/10.3390/plants10112536/s1, Table S1: Substrates in the wells of the GN2 and GP2 Biolog plates and the way they are arranged. Table S2: Isolated bacterial strains, their morphological, chemical, and biological features and identification after the Biolog system. Empty cells correspond to strains that could not be identified as the similarity index was lower than 0.5. The codes are same to those in Figure 1, Figure 2 and Figure 3 and S1–S3, where the participation of each strain in the control and treated soil samples is given. Table S3: Multivariate analysis of variance (MANOVA) for soil respiration (CO_2_ release) and bacterial abundance (of culturable strains) data from oak forest (O), mixed deciduous forest (M), riparian forest (R), phrygana (P), sandy shore (S) and desert (D) soil samples, enriched or not with fenchone, at different sampling times; EF = enrichment with fenchone, ST = sampling time; (*) significance at *p* < 0.05, (**) at *p* < 0.01, (***) at *p* < 0.001, (NS) not significant. Table S4: Multivariate analysis of variance (MANOVA) for bacterial community structure data (relative participation of the main culturable bacteria) from oak forest (O), mixed deciduous forest (M), riparian forest (R), phrygana (P), sandy shore (S) and desert (D) soil samples, enriched or not with fenchone, at different sampling times; EF = enrichment with fenchone, ST = sampling time, RP = relative participation of bacterial strains; (*) significance at *p* < 0.05, (**) at *p* < 0.01, (***) at *p* < 0.001, (NS) not significant. Table S5: Multivariate analysis of variance (MANOVA) for soil respiration (CO_2_ release) and bacterial abundance (of culturable strains) data from oak forest (O), mixed deciduous forest (M), riparian forest (R), phrygana (P), sandy shore (S) and desert (D) soil samples, enriched or not with 1,8-cineol, at different sampling times; EC = enrichment with 1,8-cineol, ST = sampling time; (*) significance at *p* < 0.05, (**) at *p* < 0.01, (***) at *p* < 0.001, (NS) not significant. Table S6: Multivariate analysis of variance (MANOVA) for bacterial community structure data (relative participation of the main culturable bacteria) of mixed deciduous forest (M), oak forest (O), riparian forest (R), phrygana (P), sandy shore (S) and desert (D) soil samples enriched or not with 1,8-cineol, at different sampling times; EC = enrichment with 1,8-cineol, ST = sampling time, RP = relative participation of bacterial strains; (*) significance at *p* < 0.05, (**) significance at *p* < 0.01, (***) significance at *p* < 0.001, (NS) not significant. Table S7: Multivariate analysis of variance (MANOVA) for soil respiration (CO_2_ release) and bacterial abundance (of culturable strains) data from oak forest (O), mixed deciduous forest (M), riparian forest (R), phrygana (P), sandy shore (S) and desert (D) soil samples, enriched or not with α-pinene, at the different sampling times; EP = enrichment with α-pinene, ST = sampling time; (*) significance at *p* < 0.05, (**) significance at *p* < 0.01, (***) significance at *p* < 0.001, (NS) not significant. Table S8: Multivariate analysis of variance (MANOVA) for bacterial community structure data (relative participation of the main culturable bacteria) from oak forest (O), mixed deciduous forest (M), riparian forest (R), phrygana (P), sandy shore (S) and desert (D) soil samples, enriched or not with α-pinene, at the different sampling times; EP = enrichment with α-pinene, ST = sampling time, RP = relative participation of bacterial strain; (*) significance at *p* < 0.05, (**) significance at *p* < 0.01, (***) significance at *p* < 0.001, (NS) not significant. Table S9: Inhibition zones (average ± se) around disks imbibed with 5, 10 and 15 μL of the monoterpenes examined for each of the 15 strains that were isolated from the soil of the ecosystems studied. Figure S1: Results regarding (a) soil respiration (average ± se), (b) abundance of the community of culturable bacteria (average ± se) and (c) relative participation of the isolated main bacterial strains in enriched with fenchone (F) and in control soil samples, (I) from a mixed deciduous (M) and (II) from a riparian (R) forest, (III) from a sandy shore (S) and (IV) from a desert (D). Fenchone was added every week. Measurements for (b) and (c) were taken at the start of the experiment and on the 4th, 7th, 10th, 13th, 16th, and 17th week; measurements for (a) started on the 4th week. At the end of 13th week, as indicated by an arrow, fenchone addition was reversed: control samples became the enriched ones and vice versa. In (a) and (b), white colour corresponds to control soil samples and grey to enriched ones. In (c), the upper row corresponds to treated samples and the lower to control samples. For the bacterial codes, see note under Table S2. Figure S2: Results regarding (a) soil respiration (average ± se), (b) abundance of the community of culturable bacteria (average ± se) and (c) relative participation of the isolated main bacterial strains in enriched with 1,8-cineol (C) and in control soil samples, (I) from a mixed deciduous (M) and (II) from a riparian (R) forest, (III) from a sandy shore (S) and (IV) from a desert (D). 1,8-Cineol was added every week. Measurements for (b) and (c) were taken at the start of the experiment and on the 4th and 7th week; measurements for (a) started on the 4th week. In (a) and (b), white colour corresponds to control soil samples and grey to enriched ones. In (c), the upper row corresponds to treated samples and the lower to control samples. For the bacterial codes, see note under Table S2. Figure S3: Results regarding (a) soil respiration (average ± se), (b) abundance of the community of culturable bacteria (average ± se) and (c) relative participation of the isolated main bacterial strains in enriched with α-pinene (P) and in control soil samples, (I) from a mixed deciduous (M) and (II) from a riparian (R) forest, (III) from a sandy shore (S) and (IV) from a desert (D). α-Pinene was added every week. Measurements for (b) and (c) were taken at the start of the experiment and on the 4th and 7th week; measurements for (a) started on the 4th week. In (a) and (b), white colour corresponds to control soil samples and grey to enriched ones. In (c), the upper row corresponds to treated samples and the lower to control samples. For the bacterial codes, see note under Table S2.
