# Supplementary material for: Monoterpene Enrichments Have Positive Impacts on Soil Bacterial Communities and the Potential of Application in Bioremediation
Source: Plants (Basel). 2021 Nov 21;10(11):2536. doi: 10.3390/plants10112536 (PMC8623845; doi:10.3390/plants10112536)
Supplement: Supplementary file 1 [file plants-10-02536-s001.zip › plants-1394670-supplementary.pdf]

SUPPLEMENT

Tables S1-S9
Figures S1-S3

Monoterpene enrichments have positive impacts on soil bacterial communities and the potential of application in bioremediation

Dimitris Chalkos[1], Katerina Karamanoli[2] and Despoina Vokou[1*]

[1] Department of Ecology, School of Biology, Aristotle University of Thessaloniki, Greece
[2] Laboratory of Agricultural Chemistry, School of Agriculture, Faculty of Agriculture Forestry and Natural Environment, Aristotle University of Thessaloniki, 54124 Thessaloniki, Greece
*Correspondence: vokou@bio.auth.gr

Table S1. Substrates in the wells of the (i) GN2 and (ii) GP2 Biolog plates, and the way they are arranged.

(i) GN2 plates

Well	Substrates											
	1	2	3	4	5	6	7	8	9	10	11	12
**A**	water	α-cyclo-dextrin	dextrin	glycogen	tween 40	tween 80	N-acetyl-D-gala-ctosamine	N-acetyl-D-glu-cosamine	adonitol	L-arabin-ose	D-arabitol	D-cello-biose
**B**	i-erythritol	D-fructose	L-fucose	D-galactose	gentio-biose	α-D-glucose	*m*-inositol	α-D-lactose	lactulose	maltose	D-mannitol	D-mannose
**C**	D-meli-biose	β-methyl-D-gluco-side	D-psicose	D-raffi-nose	L-rhamnose	D-sorbitol	sucrose	D-trehalose	turanose	xylitol	pyruvic acid methyl ester	succinic acid mono-methyl ester
**D**	acetic acid	*cis*-aconitic acid	citric acid	formic acid	D-gala-ctonic acid lactone	D-gala-cturonic acid	D-gluconic acid	D-glucos-aminic acid	D-glucu-ronic acid	α-hydroxy butyric acid	β-hydroxy butyric acid	γ-hydroxy-butyric acid
**E**	*p*-hydroxy phenyl-acetic acid	itaconic acid	α-keto-butyric acid	α-keto glutaric acid	α-keto valeric acid	D,L-lactic acid	malonic acid	propionic acid	quinic acid	D-saccharic acid	sebacic acid	succinic acid
**F**	bromo-succinic acid	succinamic acid	glucuron-amide	L-alanin-amide	D-alanine	L-alanine	L-alanyl glycine	L-aspara-gine	L-aspartic acid	L-glutamic acid	glycyl-L-aspartic acid	glycyl-L-glutamic acid
**G**	L-histidine	hydroxy-L-proline	L-leucine	L-orni-thine	L-phenyl alanine	L-proline	L-pyro-glutamic acid	D-serine	L-serine	L-threonine	D,L-carnitine	γ-amino butyric acid
**H**	urocanic acid	inosine	uridine	thymidine	phenyl-ethyl amine	putrescine	2-amino-ethanol	2,3-butane-diol	glycerol	D,L-α-glycerol phosphate	α-D-glu-cose-1-phosphate	D-glucose-6-phosphate

(ii) GP2 Plates

Well	Substrates											
	**1**	**2**	**3**	**4**	**5**	**6**	**7**	**8**	**9**	**10**	**11**	**12**
**A**	water	α-cyclo-dextrin	β-cyclo-dextrin	dextrin	glycogen	inulin	mannan	tween 40	tween 80	N-acetyl-D-glucos-amine	N-acetyl-β-D-man-nosamine	amy-gdalin
**B**	L-arabinose	D-arabitol	arbutin	D-cellobiose	D-fructose	L-fucose	D-galactose	D-galactu-ronic acid	gentio-biose	D-gluconic acid	α-D-glucose	*m*-inositol
**C**	α-D-lactose	lactulose	maltose	malto-triose	D-mannitol	D-mannose	D-melezitose	D-melibiose	α-methyl-D-galactoside	β-methyl-D-galactoside	3-methyl glucose	α-methyl-D-glucoside
**D**	β-methyl-D-glucoside	α-methyl-D-mannoside	palatinose	D-psicose	D-raffinose	L-rhamnose	D-ribose	salicin	sedo-heptulosan	D-sorbitol	stachyose	sucrose
**E**	D-tagatose	D-trehalose	turanose	xylitol	D-xylose	acetic acid	α-hydroxy-butyric acid	β-hydroxy-butyric acid	γ-hydroxy butyric Acid	*p*-hydroxy-phenyl-acetic acid	α-keto-glutaric acid	α-keto-valeric acid
**F**	lactamide	D-lactic acid methyl ester	L-lactic acid	D-malic acid	L-malic acid	pyruvatic acid methyl ester	succinic acid mono-methyl ester	propionic acid	pyruvic acid	succinamic acid	succinic acid	N-acetyl-L-glutamic acid
**G**	L-alanin-amide	D-alanine	L-alanine	L-alanyl-glycine	L-asparagine	L-glutamic acid	glycyl- L-glutamic acid	L-pyro-glutamic acid	L-serine	putrescine	2,3-butanediol	glycerol
**H**	adenosine	2'-deoxy adenosine	inosine	thymidine	uridine	adenosine-5'-mono-phosphate	thymidine-5'-mono-phosphate	uridine-5'-mono-phosphate	D-fructose-6-phosphate	α-D-glucose-1-phosphate	D-glucose-6-phosphate	D-L-α-glycerol phosphate

Table S2. Isolated bacterial strains, their morphological, chemical, and biological features and identification after the Biolog system. Empty cells correspond to strains that could not be identified as the similarity index was lower than 0.5. The codes[1] are same to those in Figs 1,2,3 and S1,S2,S3, where the participation of each strain in the control and treated soil samples is given.

Code	Gram	Shape	Catalase	Oxidase	Endospores	Molility	Colour	Identification with the Biolog system		
								**Bacterial strain**	**Probability (%)**	**Similarity index**
MF1	-	bacillus	+	+	-	-	pink	*Burkholderia glumae*	99	0.642
MF2	+	coccus	+	-	-	-	purple	*Micrococcus diversus*	99	0.583
MF3	+	bacillus	+	-	+	+	creamy white	*Brevibacterium mcbrellneri*	99	0.543
MF4	-	coccus	+	-	-	+	yellow			
MF5	-	bacillus	+	-	+	+	brown			
MF6	-	bacillus	+	-	+	+	white	*Pseudomonas caricapapayae (syringae)*	99	0.651
MF7	-	coccus	+	-	-	-	orange			
MC1	-	bacillus	+	+	-	-	pink	*Burkholderia glumae*	99	0.642
MC2	+	coccus	+	-	-	-	purple	*Micrococcus diversus*	99	0.583
MC3	+	bacillus	+	-	+	+	creamy white	*Brevibacterium mcbrellneri*	99	0.543
MC4	-	coccus	+	-	-	+	yellow			
MC5	-	bacillus	+	-	+	+	white	*Pseudomonas caricapapayae (syringae)*	99	0.651
MC6	-	coccus	+	-	-	-	orange			
MP1	-	bacillus	+	+	-	-	pink	*Burkholderia glumae*	99	0.642
MP2	+	coccus	+	-	-	-	purple	*Micrococcus diversus*	99	0.583
MP3	+	bacillus	+	-	+	+	creamy white	*Brevibacterium mcbrellneri*	99	0.543
MP4	-	coccus	+	-	-	+	yellow			
MP5	-	bacillus	+	-	+	+	white	*Pseudomonas caricapapayae (syringae)*	99	0.651
MP6	-	coccus	+	-	-	-	orange			
OF1	+	bacillus	+	-	-	-	pink	*Rhodococcus ruber*	97	0.809
OF2	+	coccus	+	-	-	-	purple	*Micrococcus diversus*	99	0.583
OF3	+	bacillus	+	-	-	+	creamy white	*Tsukamurella inchonensis*	99	0.520
OF4	-	coccus	+	-	-	+	yellow			

OF5	-	bacillus	+	-	+	+	white	*Pseudomonas caricapapayae (syringae)*	99	0.651
OF6	-	bacillus	+	-	+	+	brown			
OC1	+	bacillus	+	-	-	-	pink	*Rhodococcus ruber*	97	0.809
OC2	+	coccus	+	-	-	-	purple	*Micrococcus diversus*	99	0.583
OC3	+	bacillus	+	-	-	+	creamy white	*Tsukamurella inchonensis*	99	0.520
OC4	-	coccus	+	-	-	+	yellow			
OC5	-	bacillus	+	-	+	+	white	*Pseudomonas caricapapayae (syringae)*	99	0.651
OC6	-	coccus	+	-	-	-	orange			
OP1	+	bacillus	+	-	-	-	pink	*Rhodococcus ruber*	97	0.809
OP2	+	coccus	+	-	-	-	purple	*Micrococcus diversus*	99	0.583
OP3	+	bacillus	+	-	-	+	creamy white	*Tsukamurella inchonensis*	99	0.520
OP4	-	coccus	+	-	-	+	yellow			
OP5	-	bacillus	+	-	+	+	white	*Pseudomonas caricapapayae (syringae)*	99	0.651
OP6	-	coccus	+	-	-	-	orange			
RF1	-	bacillus	+	+	-	-	pink	*Burkholderia glumae*	99	0.642
RF2	-	coccus	+	+	+	+	purple	*Variovorax paradoxus*	94	0.505
RF3	+	coccus	+	-	-	-	creamy white	*Microccocus luteus*	99	0.578
RF4	+	coccus	+	-	-	+	yellow			
RF5	-	bacillus	+	-	+	+	white	*Pseudomonas caricapapayae (syringae)*	99	0.651
RF6	-	bacillus	+	-	+	+	brown			
RC1	-	bacillus	+	+	-	-	pink	*Burkholderia glumae*	99	0.642
RC2	-	coccus	+	+	+	+	purple	*Variovorax paradoxus*	94	0.505
RC3	+	coccus	+	-	-	-	creamy white	*Microccocus luteus*	99	0.578
RC4	+	coccus	+	-	-	+	yellow			
RC5	-	bacillus	+	-	+	+	white	*Pseudomonas caricapapayae (syringae)*	99	0.651
RC6	-	coccus	+	-	-	-	orange			
RP1	-	bacillus	+	+	-	-	pink	*Burkholderia glumae*	99	0.642
RP2	-	coccus	+	+	+	+	purple	*Variovorax paradoxus*	94	0.505
RP3	+	coccus	+	-	-	-	creamy white	*Microccocus luteus*	99	0.578
RP4	+	coccus	+	-	-	+	yellow			

RP5	-	bacillus	+	-	+	+	white	*Pseudomonas caricapapayae (syringae)*	99	0.651
RP6	-	coccus	+	-	-	-	orange			
PF1	+	bacillus	+	-	-	-	pink	*Corynebacterium nitrilophilus*	99	0.601
PF2	+	coccus	+	-	+	+	purple			
PF3	+	bacillus	+	-	-	+	creamy white	*Tsukamurella inchonensis*	99	0.520
PF4	-	bacillus	+	-	-	+	yellow	*Burkholderia gladioli*	99	0.584
PF5	-	bacillus	+	+	+	+	white	*Burkholderia glumae*	99	0.607
PF6	-	coccus	+	-	-	-	orange			
PC1	+	bacillus	+	-	-	-	pink	*Corynebacterium nitrilophilus*	99	0.601
PC2	+	coccus	+	-	+	+	purple			
PC3	+	bacillus	+	-	-	+	creamy white	*Tsukamurella inchonensis*	99	0.520
PC4	-	bacillus	+	-	-	+	yellow	*Burkholderia gladioli*	99	0.584
PC5	-	bacillus	+	+	+	+	white	*Burkholderia glumae*	99	0.607
PC6	-	coccus	+	-	-	-	orange			
PP1	+	bacillus	+	-	-	-	pink	*Corynebacterium nitrilophilus*	99	0.601
PP2	+	coccus	+	-	+	+	purple			
PP3	+	bacillus	+	-	-	+	creamy white	*Tsukamurella inchonensis*	99	0.520
PP4	-	bacillus	+	-	-	+	yellow	*Burkholderia gladioli*	99	0.584
PP5	-	bacillus	+	+	+	+	white	*Burkholderia glumae*	99	0.607
PP6	-	coccus	+	-	-	-	orange			
SF1	+	bacillus	+	-	-	+	pink			
SF2	-	bacillus	+	-	-	+	purple	*Pantoea stewartii (Erwinia)*	99	0.540
SF3	+	bacillus	+	-	-	+	creamy white	*Tsukamurella inchonensis*	99	0.520
SF4	-	coccus	+	+	-	-	yellow	*Neisseria canis*	94	0.645
SF5	-	bacillus	+	-	+	-	white			
SF6	-	coccus	+	-	-	-	orange			
SC1	+	bacillus	+	-	-	+	pink			
SC2	-	bacillus	+	-	-	+	purple	*Pantoea stewartii (Erwinia)*	99	0.540
SC3	+	bacillus	+	-	-	+	creamy white	*Tsukamurella inchonensis*	99	0.520
SC4	-	coccus	+	+	-	-	yellow	*Neisseria canis*	94	0.645

SC5	-	bacillus	+	-	+	-	white			
SC6	-	coccus	+	-	-	-	orange			
SP1	+	bacillus	+	-	-	+	pink			
SP2	-	bacillus	+	-	-	+	purple	*Pantoea stewartii (Erwinia)*	99	0.540
SP3	+	bacillus	+	-	-	+	creamy white	*Tsukamurella inchonensis*	99	0.520
SP4	-	coccus	+	+	-	-	yellow	*Neisseria canis*	94	0.645
SP5	-	bacillus	+	-	+	-	white			
SP6	-	coccus	+	-	-	-	orange			
DF1	-	bacillus	+	+	-	+	pink	*Rhizobium rhizogenes*	99	0.505
DF2	+	bacillus	+	-	-	-	purple	*Corynebacterium lypophylophlavum*	92	0.510
DF3	+	bacillus	+	-	-	+	creamy white	*Tsukamurella inchonensis*	99	0.520
DF4	-	coccus	+	+	-	-	yellow			
DF5	-	bacillus	+	-	-	+	white	*Buttiauxella gaviniae*	75	0.62
DF6	-	coccus	+	-	-	-	orange			
DC1	-	bacillus	+	+	-	+	pink	*Rhizobium rhizogenes*	99	0.505
DC2	+	bacillus	+	-	-	-	purple	*Corynebacterium lypophylophlavum*	92	0.510
DC3	+	bacillus	+	-	-	+	creamy white	*Tsukamurella inchonensis*	99	0.520
DC4	-	coccus	+	+	-	-	yellow			
DC5	-	bacillus	+	-	-	+	white	*Buttiauxella gaviniae*	75	0.62
DC6	-	coccus	+	-	-	-	orange			
DP1	-	bacillus	+	+	-	+	pink	*Rhizobium rhizogenes*	99	0.505
DP2	+	bacillus	+	-	-	-	purple	*Corynebacterium lypophylophlavum*	92	0.510
DP3	+	bacillus	+	-	-	+	creamy white	*Tsukamurella inchonensis*	99	0.520
DP4	-	coccus	+	+	-	-	yellow			
DP5	-	bacillus	+	-	-	+	white	*Buttiauxella gaviniae*	75	0.62
DP6	-	coccus	+	-	-	-	orange			

[1] The first letter of the codes corresponds to the ecosystem from which strains were isolated [mixed deciduous forest (M), oak forest (O), riparian forest (R), phrygana (P), sandy shore (S), desert (D)] and the second letter to the compound examined [fenchone (F), 1,8-cineol (C) and α-pinene (P)]; the number corresponds to each of the six (seven in the case of the mixed deciduous forest and fenchone) main bacterial strains that were isolated from the soil samples of the different treatments, which were also main strains in the control soil samples.

Table S3. Multivariate analysis of variance (MANOVA) for soil respiration (CO_2 release) and bacterial abundance (of culturable strains) data from oak forest (O), mixed deciduous forest (M), riparian forest (R), phrygana (P), sandy shore (S) and desert (D) soil samples, enriched or not with fenchone, at different sampling times; EF = enrichment with fenchone, ST = sampling time; (*) significance at $p<0.05$, (**) at $p<0.01$, (***), at $p<0.001$, (NS) not significant.

**Effect**	**df**	**Soil respiration**						**df**	**Bacterial abundance**					
		**O**	**M**	**R**	**P**	**S**	**D**		**M**	**O**	**R**	**P**	**S**	**D**
Enrichment with fenchone	1	***	***	***	***	***	***	1	***	***	**	*	***	***
Sampling time	5	***	***	***	***	***	***	6	***	***	***	***	***	***
EF x ST	5	***	***	***	***	***	***	6	***	***	***	***	***	***

Table S4. Multivariate analysis of variance (MANOVA) for bacterial community structure data (relative participation of the main culturable bacteria) from oak forest (O), mixed deciduous forest (M), riparian forest (R), phrygana (P), sandy shore (S) and desert (D) soil samples, enriched or not with fenchone, at different sampling times; EF = enrichment with fenchone, ST = sampling time, RP = relative participation of bacterial strains; (*) significance at $p<0.05$, (**) at $p<0.01$, (***), at $p<0.001$, (NS) not significant.

**Effect**	**df**	**Community structure**	**df**	**Community structure**				
		**M**		**O**	**R**	**P**	**S**	**D**
Relative participation	6	***	5	***	***	***	***	***
Enrichment with fenchone	1	NS	1	NS	NS	NS	NS	NS
RP X EF	6	***	5	***	***	***	***	***
Sampling time	6	NS	6	NS	NS	NS	NS	NS
ST X RP	36	***	30	***	***	***	***	***
ST X EF	6	NS	6	NS	NS	NS	NS	NS
ST X RP X EF	36	***	30	***	***	***	***	***

Table S5. Multivariate analysis of variance (MANOVA) for soil respiration (CO_2 release) and bacterial abundance (of culturable strains) data from oak forest (O), mixed deciduous forest (M), riparian forest (R), phrygana (P), sandy shore (S) and desert (D) soil samples, enriched or not with 1,8-cineol, at different sampling times; EC = enrichment with 1,8-cineol, ST = sampling time; (*) significance at $p<0.05$, (**) at $p<0.01$, (***), at $p<0.001$, (NS) not significant.

Effect	df	Soil respiration						df	Bacterial abundance					
		O	M	R	P	S	D		M	O	R	P	S	D
Enrichment with 1,8-cineol	1	***	***	***	***	***	***	1	***	*	**	**	**	**
Sampling time	1	NS	***	NS	***	***	***	2	***	***	***	***	***	***
EC x ST	1	NS	*	NS	***	***	**	2	***	**	***	***	***	***

Table S6. Multivariate analysis of variance (MANOVA) for bacterial community structure data (relative participation of the main culturable bacteria) of mixed deciduous forest (M), oak forest (O), riparian forest (R), phrygana (P), sandy shore (S) and desert (D) soil samples enriched or not with 1,8-cineol, at different sampling times; EC = enrichment with 1,8-cineol, ST = sampling time, RP = relative participation of bacterial strains; (*) significance at $p<0.05$, (**) significance at $p<0.01$, (***) significance at $p<0.001$, (NS) not significant.

Effect	df	Community structure					
		O	M	R	P	S	D
Relative participation	5	***	***	***	***	***	***
Enrichment with 1,8-cineol	1	NS	NS	NS	NS	NS	NS
RP X EC	5	***	***	***	***	***	***
Sampling time	2	NS	NS	NS	NS	NS	NS
ST X RP	10	***	***	***	***	***	***
ST X EC	2	NS	NS	NS	NS	NS	NS
ST X RP X EC	10	***	***	***	***	***	***

Table S7. Multivariate analysis of variance (MANOVA) for soil respiration (CO_2 release) and bacterial abundance (of culturable strains) data from oak forest (O), mixed deciduous forest (M), riparian forest (R), phrygana (P), sandy shore (S) and desert (D) soil samples, enriched or not with α-pinene, at the different sampling times; EP = enrichment with α-pinene, ST = sampling time; (*) significance at p<0.05, (**) significance at p<0.01, (***) significance at p<0.001, (NS) not significant.

Effect	df	Soil respiration						df	Bacterial abundance					
		O	M	R	P	S	D		O	M	R	P	S	D
Enrichment with α-pinene	1	***	***	***	***	***	***	1	***	***	**	*	**	***
Sampling time	1	NS	NS	*	**	**	***	2	***	***	***	**	***	***
EP x ST	1	NS	NS	NS	*	NS	**	2	***	***	***	*	***	***

Table S8. Multivariate analysis of variance (MANOVA) for bacterial community structure data (relative participation of the main culturable bacteria) from oak forest (O), mixed deciduous forest (M), riparian forest (R), phrygana (P), sandy shore (S) and desert (D) soil samples, enriched or not with α-pinene, at the different sampling times; EP = enrichment with α-pinene, ST = sampling time, RP = relative participation of bacterial strains; (*) significance at p<0.05, (**) significance at p<0.01, (***) significance at p<0.001, (NS) not significant.

Effect	df	Community structure					
		O	M	R	P	S	D
Relative participation	5	***	***	***	***	***	***
Enrichment with α-pinene	1	NS	NS	NS	NS	NS	NS
RP X EP	5	***	***	***	***	***	***
Sampling time	2	NS	NS	NS	NS	NS	NS
ST X RP	10	***	***	***	***	***	***
ST X EP	2	NS	NS	NS	NS	NS	NS
ST X RP X EP	10	***	***	***	***	***	***

Table S9. Inhibition zones (average ± se) around disks imbibed with 5, 10 and 15 μl of the monoterpenes examined for each of the 15 strains that were isolated from the soil of the ecosystems studied.

Compound	Quantity (μl)	Inhibition zone (mm)						
		Gram positive strains						
		Brevibacterium mcbrellneri	*Corynebacterium lypophylophlavum*	*C. nitrilophilus*	*Micrococcus diversus*	*M. luteus*	*Rhodococcus ruber*	*Tsukamurella inchonensis*
Fenchone	5	0	0	0	0	5±1	0	0
	10	0	0	0	0	7±1	0	8±2
	15	0	0	0	0	8±2	0	15±3
1.8-Cineol	5	4±1	0	0	0	0	0	0
	10	7±1	0	0	0	0	0	0
	15	9±1	0	0	0	10±3	0	8±2
α-Pinene	5	0	0	0	0	0	0	0
	10	0	0	0	0	0	0	0
	15	0	0	0	0	0	0	0

Compound	Quantity (μl)	Gram negative strains							
		Burkholderia gladioli	*B. glumae*	*Buttiauxella gaviniae*	*Neisseria canis*	*Pantoea stewartii*	*Pseudomonas caricapapaya*	*Rhizobium rhizogenes*	*Variovorax paradoxus*
Fenchone	5	12±2	0	3±1	0	0	35±4	6±1	0
	10	20±3	0	5±1	0	0	90±0	13±2	0
	15	25±3	0	9±2	0	0	90±0	18±2	0
1,8-Cineol	5	0	0	6±1	3±1	0	20±4	0	5±1
	10	5±1	0	11±2	7±1	0	45±5	12±4	12±3
	15	9±2	0	17±2	13±2	0	52±7	22±6	19±4
α-Pinene	5	0	0	0	0	0	0	0	0
	10	0	0	0	0	0	0	0	0
	15	0	0	0	0	0	0	0	0

(I)

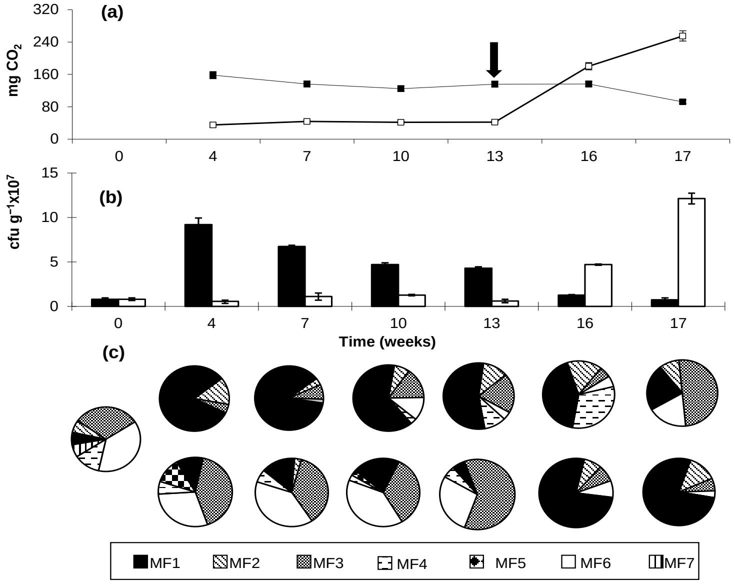

(a)
mg CO2
320
240
160
80
0
0
4
7
10
13
16
17
(b)
cfu g-1x10^7
15
10
5
0
Time (weeks)
(c)
MF1
MF2
MF3
MF4
MF5
MF6
MF7

(II)

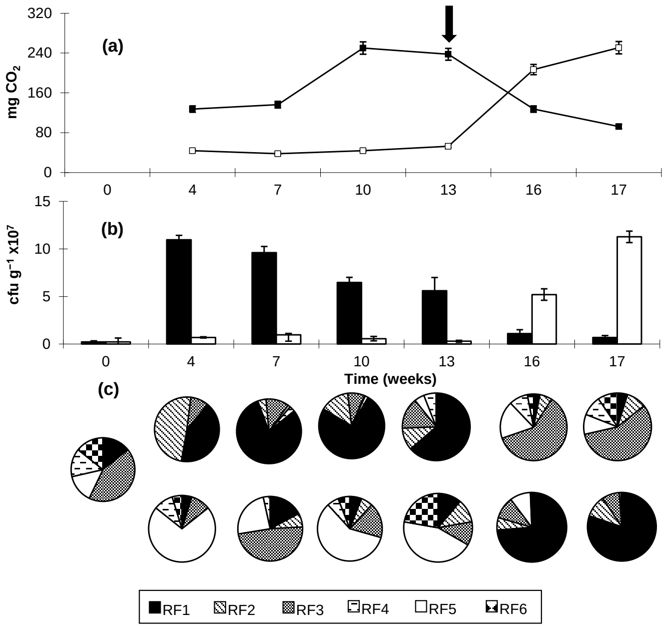

(a)
mg CO2
320
240
160
80
0
0
4
7
10
13
16
17
(b)
cfu g-1 x10^7
15
10
5
0
Time (weeks)
(c)
RF1
RF2
RF3
RF4
RF5
RF6

(III)

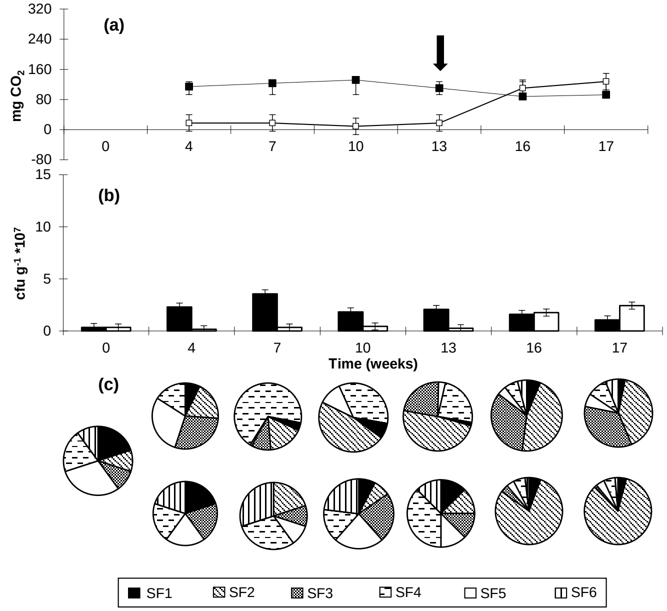

(a)
mg CO2
320
240
160
80
0
-80
0
4
7
10
13
16
17
(b)
cfu g-1 *10^7
15
10
5
0
Time (weeks)
(c)
SF1
SF2
SF3
SF4
SF5
SF6

(IV)

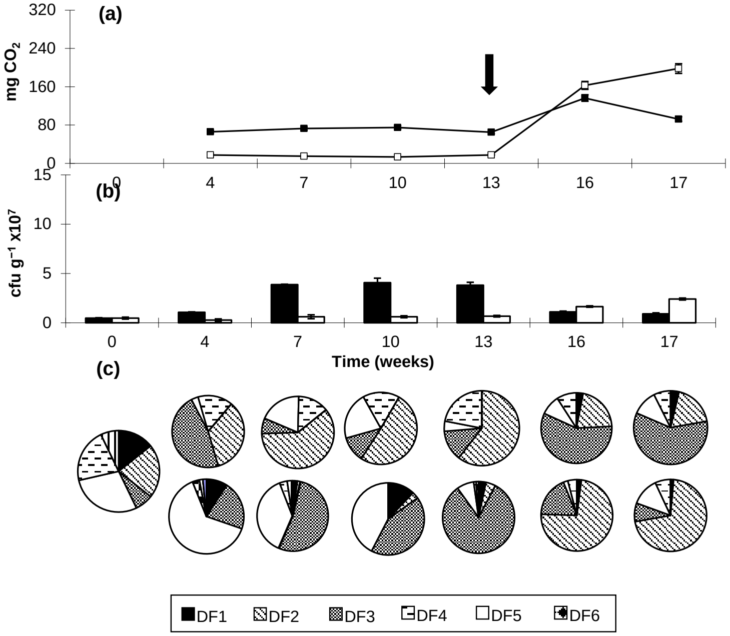

(a)
mg CO2
320
240
160
80
0
0
4
7
10
13
16
17
(b)
cfu g-1 x10^7
15
10
5
0
Time (weeks)
(c)
DF1
DF2
DF3
DF4
DF5
DF6

Figure S1: Results regarding (a) soil respiration (average ± se), (b) abundance of the community of culturable bacteria (average ± se) and (c) relative participation of the isolated main bacterial strains in enriched with fenchone (F) and in control soil samples, (I) from a mixed deciduous (M) and (II) from a riparian (R) forest, (III) from a sandy shore (S) and (IV) from a desert (D). Fenchone was added every week. Measurements for (b) and (c) were taken at the start of the experiment and on the 4th, 7th, 10th, 13th, 16th, and 17th week; measurements for (a) started on the 4th week. At the end of 13th week, as indicated by an arrow, fenchone addition was reversed: control samples became the enriched ones and *vice versa*. In (a) and (b), white colour corresponds to control soil samples and grey to enriched ones. In (c), the upper row corresponds to treated samples and the lower to control samples. For the bacterial codes, see note under Table S2.

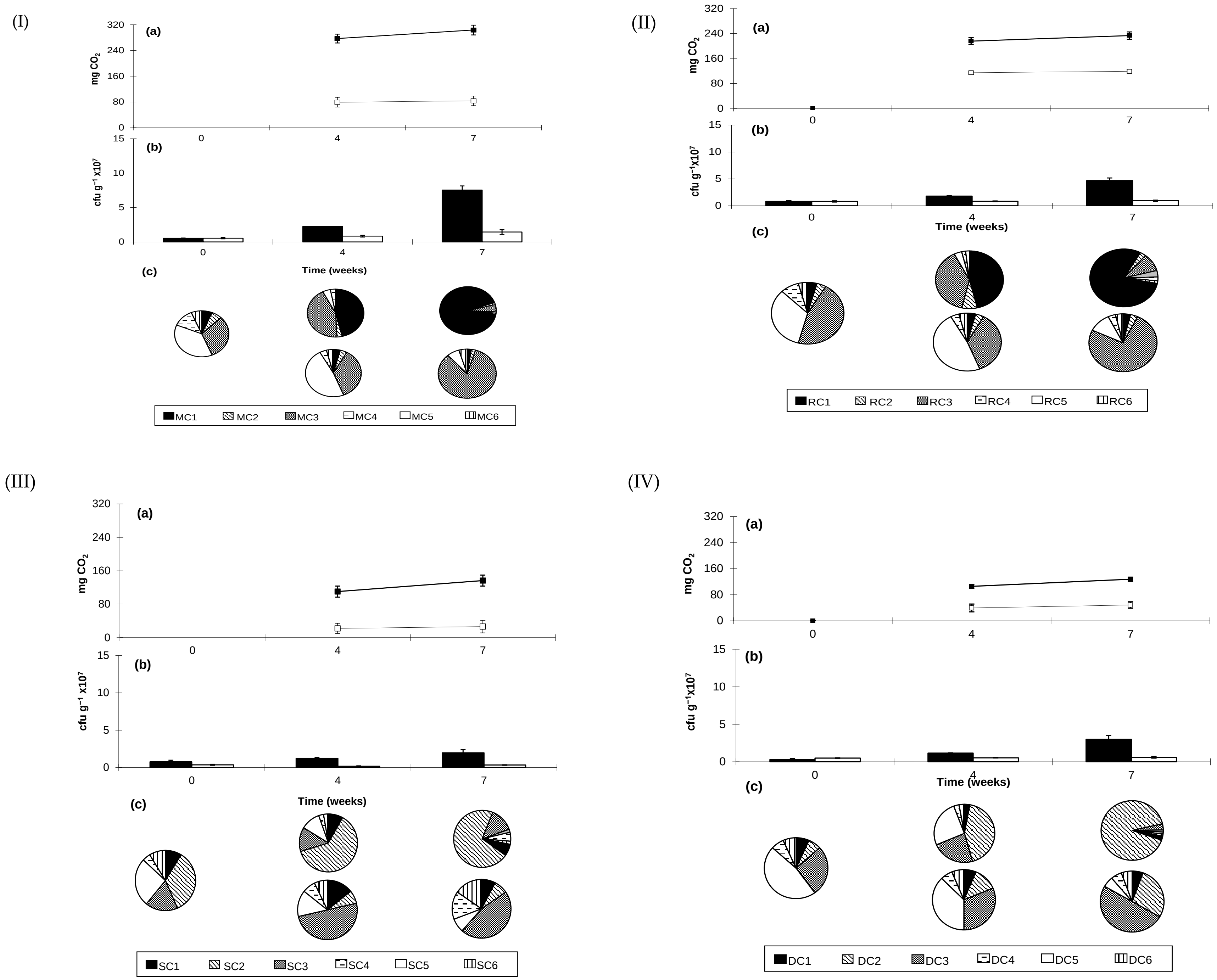

(I)
(a)
mg CO_2
320
240
160
80
0
0
4
7
(b)
cfu g⁻¹ x10⁷
15
10
5
0
Time (weeks)
(c)
MC1 MC2 MC3 MC4 MC5 MC6
(II)
(a)
mg CO_2
(b)
cfu g⁻¹ x10⁷
Time (weeks)
(c)
RC1 RC2 RC3 RC4 RC5 RC6
(III)
(a)
mg CO_2
(b)
cfu g⁻¹ x10⁷
Time (weeks)
(c)
SC1 SC2 SC3 SC4 SC5 SC6
(IV)
(a)
mg CO_2
(b)
cfu g⁻¹ x10⁷
Time (weeks)
(c)
DC1 DC2 DC3 DC4 DC5 DC6

Figure S2: Results regarding (a) soil respiration (average ± se), (b) abundance of the community of culturable bacteria (average ± se) and (c) relative participation of the isolated main bacterial strains in enriched with 1,8-cineol (C) and in control soil samples, (I) from a mixed deciduous (M) and (II) from a riparian (R) forest, (III) from a sandy shore (S) and (IV) from a desert (D). 1,8-Cineol was added every week. Measurements for (b) and (c) were taken at the start of the experiment and on the 4th and 7th week; measurements for (a) started on the 4th week. In (a) and (b), white colour corresponds to control soil samples and grey to enriched ones. In (c), the upper row corresponds to treated samples and the lower to control samples. For the bacterial codes, see note under Table S2.

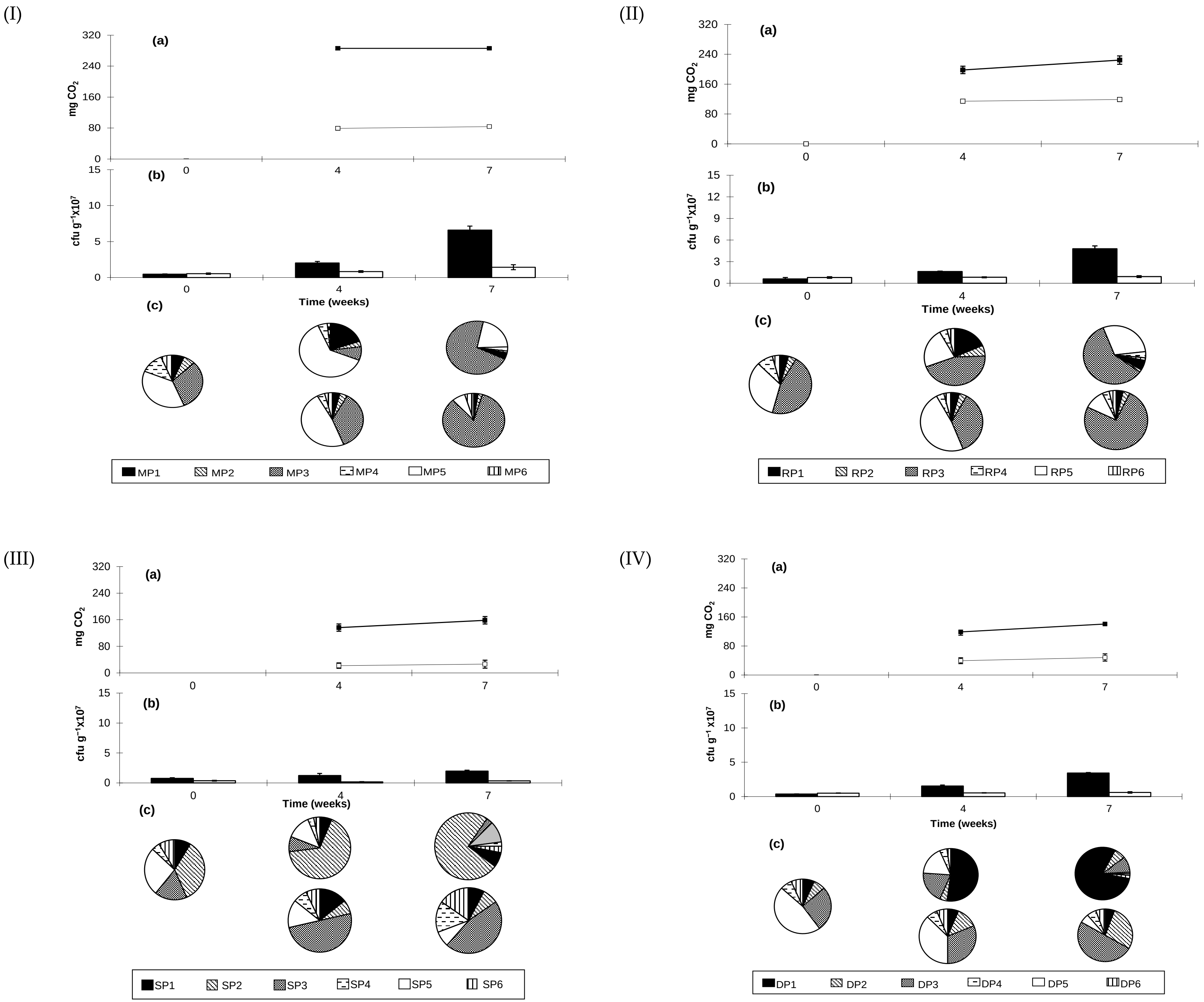
(I)
(a)
320
240
160
80
0
mg CO_2
0
4
7
15
(b)
10
5
0
cfu g^{-1}x10^7
0
4
7
Time (weeks)
(c)
MP1
MP2
MP3
MP4
MP5
MP6
(II)
(a)
320
240
160
80
0
mg CO_2
0
4
7
15
12
9
6
3
0
(b)
cfu g^{-1}x10^7
0
4
7
Time (weeks)
(c)
RP1
RP2
RP3
RP4
RP5
RP6
(III)
320
240
160
80
0
(a)
mg CO_2
0
4
7
15
10
5
0
(b)
cfu g^{-1}x10^7
0
4
7
Time (weeks)
(c)
SP1
SP2
SP3
SP4
SP5
SP6
(IV)
320
240
160
80
0
(a)
mg CO_2
0
4
7
15
10
5
0
(b)
cfu g^{-1} x10^7
0
4
7
Time (weeks)
(c)
DP1
DP2
DP3
DP4
DP5
DP6

Figure S3: Results regarding (a) soil respiration (average ± se), (b) abundance of the community of culturable bacteria (average ± se) and (c) relative participation of the isolated main bacterial strains in enriched with α-pinene (P) and in control soil samples, (I) from a mixed deciduous (M) and (II) from a riparian (R) forest, (III) from a sandy shore (S) and (IV) from a desert (D). α-Pinene was added every week. Measurements for (b) and (c) were taken at the start of the experiment and on the 4th and 7th week; measurements for (a) started on the 4th week. In (a) and (b), white colour corresponds to control soil samples and grey to enriched ones. In (c), the upper row corresponds to treated samples and the lower to control samples. For the bacterial codes, see note under Table S2.
